# Integral nursing leadership and its association with job satisfaction and turnover intention among nurses: a cross-sectional study

**DOI:** 10.1038/s41598-026-51285-5

**Published:** 2026-05-02

**Authors:** Fatemeh Dadashzadeh, Saeid Mehri, Alireza Mirzaei, Aghil Habibi Soola

**Affiliations:** Department of Emergency Nursing, School of Nursing and Midwifery, Ardabil University of Medical Sciences, Ardabil, 615751147 Iran

**Keywords:** Integral nursing leadership, Job satisfaction, Turnover intention, Nursing workforce, Integral nursing leadership scale (INLS), Health care, Health occupations, Medical research

## Abstract

Integral nursing leadership, based on Wilber’s Integral Framework, is vital for fostering positive work environments and aligning staff with organizational goals. This study assessed the status of integral nursing leadership and its association with job satisfaction and turnover intention among nurses in Ardabil, Iran. This cross-sectional correlational study was conducted from November 2024 to January 2025 with 450 nurses from five hospitals affiliated with Ardabil University of Medical Sciences. Data were collected using a demographic form, the Integral Nursing Leadership Scale (INLS), and single-item measures for job satisfaction and turnover intention. Analysis was performed in SPSS-26 using descriptive statistics, Pearson correlation, and linear regression (*p* < 0.05). Job satisfaction showed a significant, strong negative correlation with turnover intention (*r* = -0.585, *p* < 0.01). The mean score for integral nursing leadership was 3.85 out of 6. Only 37.3% of nurses reported job satisfaction, while 62.7% were dissatisfied and intended to leave. Regression analysis indicated that integral nursing leadership was a significant positive predictor of job satisfaction (B = 0.253, *p* < 0.001) and a significant negative predictor of turnover intention (B = -0.193, *p* < 0.001). Higher levels of integral nursing leadership are associated with increased job satisfaction and reduced turnover intention among nurses. This highlights the importance of developing integral leadership qualities to improve workplace culture and retention. Future research should investigate longitudinal effects and develop targeted interventions based on these findings.

## Introduction

Nursing leadership is a fundamental responsibility that transcends the roles of managers and directors; it is essential for all healthcare professionals to embrace leadership in order to align personnel with organizational goals^1^. Effective nurse leadership fosters a positive work environment, which is crucial for delivering safe, high-quality patient care and achieving institutional outcomes^2^.

While prevailing paradigms such as transformational leadership emphasize relational aspects^3^, integral leadership offers a more comprehensive and humanistic framework. This approach advocates for a holistic understanding of leadership’s multifaceted dimensions^4^ and represents a synergistic combination of functions such as task coordination, relational management, and systemic integration^5^. Integral leadership highlights that leadership extends beyond managers, involving all professionals in shaping the work environment and impacting care outcomes^6,7^. Grounded in Wilber’s integral model, it examines leadership through four interdependent quadrants: the individual’s interior experience (“I”), observable behaviors (“It”), shared culture (“We”), and organizational systems (“Its”). This model ensures a well-rounded perspective by integrating subjective and objective, individual and collective dimensions^8,9^.

In healthcare settings, leadership style significantly impacts employee performance^10^. Effective leadership and supportive human resource practices are vital to reducing turnover, enhancing commitment, and fostering a stable work environment^11^. Furthermore, strong nurse leadership can mitigate burnout a common challenge in the profession^12^. Given that nurses are vital organizational resources, ensuring their retention and satisfaction is critical to institutional success^11^.

Many nurses leave the profession due to low job satisfaction, heavy workloads, and limited autonomy^13^. Job dissatisfaction contributes to high turnover, which in turn exacerbates the workload of remaining staff, creating a cycle of attrition^14^. Research in various contexts, such as Greek and Finnish hospitals, confirms that leadership dynamics significantly affect job satisfaction, underscoring the need for effective leadership styles among nursing managers^15,16^. Equipping leaders with essential skills can improve team cohesion and productivity^17^.

The relationship between job satisfaction and leadership underscores the importance of cultivating a work environment focused on employee well-being^18^. When staff feel valued, satisfaction increases, leading to greater engagement and reduced turnover^19^. In light of persistent global nursing shortages^20^, nurse leaders must demonstrate strong competencies to maximize staff potential and meet organizational objectives^21^.

This study aims to evaluate the current state of integral nursing leadership and examine its relationship with job satisfaction and turnover intention among nurses in Ardabil. By addressing this gap, the research seeks to enhance understanding of how integral leadership influences nurse outcomes and contributes to high-quality healthcare. The findings are expected to provide valuable insights for creating supportive work environments, increasing engagement, and reducing turnover, thereby promoting a more stable and productive healthcare workforce.

## Methods

### Design

A cross-sectional, correlational design was employed.

### Setting and sample

The study was conducted across five teaching hospitals affiliated with Ardabil University of Medical Sciences. The sample included clinical nurses who were willing to participate and had at least six months of work experience. Nurses on long-term leave (e.g., maternity, extended sick leave) or those holding formal leadership positions were excluded to minimize bias. Individuals with a history of hearing impairment, neurological, or psychiatric conditions were also excluded, as were incomplete questionnaires.

The sample size was calculated using G*Power 3.1 software. Based on a multiple linear regression model with 8 predictors (demographic variables and leadership scores), an anticipated small-to-medium effect size (f² = 0.04), an alpha of 0.05, and a power of 80%, a minimum of 416 participants was required. To account for potential non-response or incomplete data, the sample was increased to 450.

A proportional sampling method was used. First, a list of eligible nurses from all five hospitals was compiled. Participants were then randomly selected from each hospital based on its proportion of the total nursing workforce. The final distribution was: Imam Khomeini Hospital (*n* = 209), Fatemi Hospital (*n* = 79), Imam Reza Hospital (*n* = 38), Alavi Hospital (*n* = 59), and Bu Ali Hospital (*n* = 66). Questionnaires were distributed in coordination with hospital nursing offices. Any selected nurse who did not meet the inclusion criteria or declined to participate was replaced by another randomly selected nurse from the same hospital.

### Data collection

Data were collected between November 2024 and January 2025 using a self-administered questionnaire packet consisting of four sections: a demographic form, the Integral Nursing Leadership Scale (INLS), and single-item measures for job satisfaction and turnover intention.

Instruments

1. Demographic Information Form: This form captured data on age, gender, marital status, work experience, education level, clinical unit, monthly overtime hours, shift rotation pattern, and self-reported health status.
2. Integral Nursing Leadership Scale (INLS): The 30-item INLS, developed and validated by Cho and Choi^6^, was used to measure perceived integral leadership. It comprises four subscales: Individual Leadership Qualities (9 items), Individual Performance (9 items), Influencing Organizational Culture (7 items), and Organizational Excellence (5 items). Responses are recorded on a 6-point Likert scale (1 = Strongly Disagree to 6 = Strongly Agree), with higher average scores indicating more positive perceptions of integral leadership. The original scale demonstrated excellent reliability (Cronbach’s α = 0.97) and validity^6^.
  For use in this study, the INLS was translated into Persian using a standard forward-backward translation procedure by two independent bilingual experts. Content validity was assessed by a panel of 12 nursing faculty members, yielding favorable indices (CVR = 0.92, CVI = 0.95). In the present sample, the scale showed exceptional internal consistency (Cronbach’s α = 0.98).
3. Job Satisfaction and Turnover Intention: Both constructs were assessed using single-item, 5-point Likert scale measures, a method established as efficient and valid in large-scale surveys^22^.

- Job Satisfaction: Participants responded to “How satisfied are you with your job overall?” (1 = Very Dissatisfied to 5 = Very Satisfied).
- Turnover Intention: Participants selected the statement best reflecting their intention from: 1 (“I never think about leaving this organization”) to 5 (“I am actively looking for another job and will leave when I receive an offer”).

### Data analysis

Data were analyzed using IBM SPSS Statistics (Version 26.0). Descriptive statistics (frequencies, percentages, means, standard deviations) summarized all variables. The Kolmogorov-Smirnov test was used to assess the normality of continuous variables (integral leadership, job satisfaction, turnover intention) to inform the choice of subsequent parametric tests. Relationships among the main study variables were examined using Pearson’s correlation coefficients. To identify predictors of job satisfaction and turnover intention, two separate multiple linear regression analyses were performed. In these models, demographic variables were entered in the first block as control variables, and the total INLS score was entered in the second block to examine its unique predictive contribution. Assumptions of linear regression (linearity, independence of errors, homoscedasticity, and absence of multicollinearity) were checked and met.

### Ethical considerations

Ethical approval was granted by the Research Ethics Committee of Ardabil University of Medical Sciences (IR.ARUMS.REC.1403.222). The study adhered to the principles of the Declaration of Helsinki. Participants received written and verbal information about the study aims and procedures. Written informed consent was obtained from all participants. Participation was voluntary, with the right to withdraw at any time without consequence. Confidentiality was maintained through anonymous data collection and secure storage, with access restricted to the research team.

## Results

In this study, 450 nurses participated, with more than half being female (*n* = 258, 57.3%). The average age was 30.14 years (SD = 5.97). Nearly half of the participants (*n* = 251, 55.8%) were single, and a significant majority held a bachelor’s degree in nursing (*n* = 395, 87.8%). The demographic and occupational characteristics of the participants are summarized in Table 1.

**Table 1:** Demographic and Occupational Characteristics of Participants (*n* = 450).

Variable	Categories	*n* (%) or mean ± SD
Age (years)		30.14 ± 5.97
Gender	Male	192 (42.7%)
	Female	258 (57.3%)
Marital Status	Single	251 (55.8%)
	Married	199 (44.2%)
Education Level	Bachelor’s degree	395 (87.8%)
	Master’s degree and higher	55 (12.2%)
Clinical Unit	Emergency department	119 (26.4%)
	General inpatient department	87 (19.3%)
	Surgical inpatient department	65 (14.4%)
	Pediatric department	27 (6.0%)
	Obstetrics & gynecology department	17 (3.8%)
	Intensive care unit	31 (6.9%)
	Operating room	23 (5.1%)
	Others	81 (18.0%)
Monthly Overtime (hours)	< 40	104 (23.1%)
	40–80	213 (47.3%)
	80–120	99 (22.0%)
	> 120	34 (7.6%)
Work Shift Rotation	Morning	97 (21.6%)
	Evening	197 (43.8%)
	Night	156 (34.7%)
Self-reported Health	Poor	68 (15.1%)
	Average	180 (40.0%)
	Good	202 (44.9%)
Number of Beds in Unit		23.29 ± 12.99
Total Work Experience (years)		6.18 ± 5.45
Experience in Current Dept. (years)		4.03 ± 3.68

The mean scores for integral nursing leadership, job satisfaction, and turnover intention are presented in Table 2. The mean integral nursing leadership score was 3.85 (SD = 1.35) on a 6-point scale. Using a cut-off score of ≥ 3 on the 5-point scale to indicate satisfaction, 37.3% (*n* = 168) of participants reported job satisfaction (Mean = 2.74, SD = 1.29), while 62.7% (*n* = 282) expressed dissatisfaction and reported an intention to leave (Mean for turnover intention = 2.54, SD = 1.21).

**Table 2:** Descriptive statistics for main study variables (*n* = 450).

Variable	Mean	SD	Possible Range
Integral Nursing Leadership	3.85	1.35	1–6
Job Satisfaction	2.74	1.29	1–5
Turnover Intention	2.54	1.21	1–5

Correlation analysis revealed significant relationships among the key variables (Table 3). Integral nursing leadership showed a significant positive correlation with job satisfaction (*r* = 0.390, *p* < 0.01) and a significant negative correlation with turnover intention (*r* = -0.349, *p* < 0.01). As expected, job satisfaction and turnover intention were strongly negatively correlated (*r* = -0.585, *p* < 0.01).

**Table 3:** Correlation matrix of main study variables (*n* = 450).

Variable	1	2	3
1. Integral Nursing Leadership	1		
2. Job Satisfaction	0.390**	1	
3. Turnover Intention	-0.349**	-0.585**	1
Note: **p < 0.01 (two-tailed).			

The results of the linear regression analyses are presented in Table 4. After controlling for demographic and occupational variables, integral nursing leadership was a significant predictor for both outcomes. It was a positive predictor of job satisfaction (B = 0.253, *p* < 0.001) and a negative predictor of turnover intention (B = -0.193, *p* < 0.001). Better self-reported health (using “Good” as the reference category) was a significant positive predictor of job satisfaction (B = 0.400, *p* < 0.001) and a significant negative predictor of turnover intention (B = -0.525, *p* < 0.001). This indicates that nurses reporting “Poor” or “Average” health had lower job satisfaction and higher turnover intention compared to those reporting “Good” health. Working more overtime hours was a significant negative predictor of job satisfaction (B = -0.164, *p* = 0.021) and a positive predictor of turnover intention (B = 0.128, *p* = 0.042). Working in units with more than 20 beds was a significant negative predictor of job satisfaction (B = -0.009, *p* = 0.045). The regression models explained 26.5% of the variance in job satisfaction (F = 13.144, *p* < 0.001) and 23.4% of the variance in turnover intention (F = 11.126, *p* < 0.001).

**Table 4:** Linear Regression Analysis Predicting Job Satisfaction and Turnover Intention (*n* = 450).

Predictor	Job Satisfaction		Turnover Intention	
	B (SE)	*p*-value	B (SE)	*p*-value
**(Constant)**	1.712 (0.764)	0.026	3.515 (0.736)	< 0.001
Age (years)	-0.010 (0.022)	0.648	0.022 (0.021)	0.299
Gender (Ref: Female)	-0.181 (0.119)	0.130	0.000 (0.115)	0.998
Marital Status (Ref: Single)	0.205 (0.131)	0.117	-0.250 (0.126)	0.048*
Education (Ref: Bachelor’s)	0.242 (0.168)	0.151	0.016 (0.162)	0.920
Overtime Hours (Ref: <40 h)	**-0.164 (0.071)**	0.021*	**0.128 (0.068)**	0.042*
Work Shift (Ref: Morning)	-0.193 (0.077)	0.512	0.118 (0.074)	0.112
Health Status (Ref: Good)	**0.400 (0.086)**	< 0.001*	**-0.525 (0.082)**	< 0.001*
Unit Bed Count (> 20 beds)	**-0.009 (0.004)**	0.045*	0.007 (0.004)	0.104
**Integral Nursing Leadership**	**0.253 (0.044)**	< 0.001*	**-0.193 (0.042)**	< 0.001*
**Model Summary**	**R² = 0.265**,** F = 13.144**,** p < 0.001**		**R² = 0.234**,** F = 11.126**,** p < 0.001**	

B = Unstandardized Coefficient; SE = Standard Error. * *p* < 0.05. For categorical predictors, the reference category is indicated in parentheses. Control variables not reaching significance in either model (Unit type, Total work experience, Experience in current department) are omitted from this table for clarity but were included in the full analysis.

## Discussion

Nursing staff are pivotal resources within healthcare institutions, and their retention is critical, especially in regions facing nursing shortages^11,21^. This study examined the relationship between integral nursing leadership, job satisfaction, and turnover intention among nurses in Ardabil, Iran. The findings underscore the significant role of leadership style in shaping workforce stability.

The average integral nursing leadership score of 3.85 (out of 6) indicates a moderate level of perceived leadership competence, with clear potential for enhancement. This score is notably higher than those reported in studies focusing on more limited leadership styles, such as transactional leadership^23^, suggesting that the integral framework may encompass a broader range of positive managerial behaviors. However, the concurrent finding that only 37.3% of nurses were satisfied with their jobs, while 62.7% reported an intention to leave, reveals a critical disconnect. This situation highlights an urgent need for healthcare organizations to strategically develop leadership capabilities not merely to improve scores on a scale, but to meaningfully impact the work environment and employee experience.

The significant positive association between integral nursing leadership and job satisfaction aligns with established literature linking constructive leadership to staff morale. For instance, similar correlations have been observed with transformational and authentic leadership styles^17,24^. The integral model’s distinct contribution may lie in its holistic mandate, which requires leaders to attend simultaneously to individual competencies, team culture, and organizational systems^6,25^. This comprehensive focus could make it particularly effective in addressing the multi-faceted sources of nurse dissatisfaction. Our regression analysis confirmed that integral leadership was a significant positive predictor of job satisfaction, even after controlling for key demographic variables.

Furthermore, integral nursing leadership demonstrated a significant negative correlation with turnover intention. This finding consolidates evidence from reviews showing that positive, engaging leadership styles are consistently associated with reduced desire to leave^26,27^. In contrast, passive or authoritarian styles exacerbate turnover intentions^26^. The integral approach, by promoting empowerment, collaboration, and systemic awareness, may help mitigate specific drivers of turnover such as feelings of disempowerment, poor communication, and unsupportive unit culture.

The strong negative correlation between job satisfaction and turnover intention (*r* = -0.585) reaffirms satisfaction as a core, proximal determinant of retention^28^. This robust relationship suggests that job satisfaction often acts as a key mediator through which other organizational factors, including leadership, ultimately influence a nurse’s decision to stay or leave^29,30^. Therefore, interventions aimed at improving job satisfaction are likely to have a direct and powerful effect on retention.

When comparing our job satisfaction and turnover intention figures with other studies, meaningful interpretation requires caution. While our satisfaction rate appears higher than in some earlier Iranian studies^22^, it remains substantially lower than rates reported in certain European contexts^13,31^. Similarly, the high turnover intention observed is comparable to rates in some healthcare systems under strain^27^ but differs from others. These variations likely reflect profound differences in healthcare infrastructure, workload, economic conditions, and cultural values. Such comparisons highlight that while leadership is a universal factor, its implementation and impact are inevitably moderated by local contextual factors. Therefore, the generalizability of specific numerical benchmarks is limited.

Beyond leadership, several contextual and demographic factors in our study emerged as significant. Nurses with better self-reported health reported higher satisfaction and lower turnover intention, reinforcing the bidirectional link between well-being and work engagement^32,33^. Conversely, increased overtime hours negatively impacted satisfaction and increased turnover intention, echoing global concerns over burnout and unsustainable workloads^34,35^. The finding that working in units with more beds predicted lower satisfaction further points to workload and patient acuity as chronic stressors^36,37^. These factors collectively paint a picture of a work environment where physical and mental strain, often stemming from structural and staffing challenges, significantly undermines morale and retention.

### Implications for nursing practice

The findings of this study highlight the critical importance of strengthening integral nursing leadership, managing workload, and promoting nurses’ health and job satisfaction to improve retention and patient care quality in clinical settings. Healthcare organizations should prioritize leadership development programs that foster positive styles, such as transformational and participative approaches, while addressing factors like overtime, workload, and nurses’ well-being. Implementing supportive policies and continuous professional development can create a more satisfying work environment, reducing turnover and enhancing the overall safety and effectiveness of patient care.

### Limitations

This study has several limitations that warrant acknowledgment. First, the data for the study variables were collected through self-report measures from nurses, which may introduce biases such as social desirability or recall bias. Specifically, job satisfaction and turnover intention were assessed with single-item tools, which may not fully capture these complex constructs; employing multi-item validated scales in future research could provide greater reliability and depth. Although participants were encouraged to provide accurate responses by emphasizing the importance of the research objectives, these measurement limitations remain. Second, there was a potential risk of non-cooperation from participants, as participation was voluntary; efforts to enhance cooperation included clear communication of the study’s aims and potential benefits. Lastly, the sample primarily comprised young, bachelor’s-prepared nurses from Ardabil University of Medical Sciences, which may limit the generalizability of the findings to broader nursing populations with different demographics, educational backgrounds, and healthcare contexts. Future studies should aim to include more diverse and representative samples from various regions and settings, as well as adopt more comprehensive measurement tools to improve external validity.

## Conclusion

This study emphasizes the critical role of integral nursing leadership in enhancing job satisfaction and reducing turnover among nursing staff. To achieve these outcomes, healthcare organizations should prioritize the development of targeted leadership training programs that focus on building leadership competencies, effective communication, and empowering nursing staff. Additionally, addressing key demographic factors such as health status and working conditions within these programs can create a more supportive and tailored environment. The findings suggest that strong nursing leadership fosters a positive workplace culture, leading to higher job satisfaction and lower turnover intentions. To strengthen these insights, future research should include longitudinal or experimental studies to validate and expand upon these results, exploring how leadership interventions impact nurse retention over time. Furthermore, designing tailored leadership strategies that consider the diverse needs of nursing populations can enhance retention efforts and organizational outcomes. Ultimately, implementing structured leadership development initiatives and conducting further rigorous research will benefit healthcare organizations and their workforce sustainability.

## Data Availability

The datasets generated and/or analysed during the current study are available from the corresponding [A. HS] author upon reasonable request.
